# Complete occlusion of an ascending colon varix and its contributors using endoscopic cyanoacrylate glue injection

**DOI:** 10.1055/a-2227-3499

**Published:** 2024-01-17

**Authors:** Anastasios C. Manolakis, Dimitrios Chougias, Stelios Kyriakidis, Ioannis Hatzoudis, Eirini Deligianni, Georgios Gelas, Andreas Kapsoritakis

**Affiliations:** 1 University of Thessaly School of Medicine, Larissa, Greece; 2 69176 Department of Gastroenterology, General University Hospital of Larissa, Larissa, Greece

Bleeding from colonic varices is a rare occurrence 1 , lacking uniform management and usually treated with radiology interventions at the expense of hepatic encephalopathy aggravation 2 . Endoscopic cyanoacrylate glue injection is used for gastric variceal bleeding 3 , however its use for ascending colon varices is hindered by the complexity of collaterals, scarcity of cases, cumbersome accessibility, injection needle occlusion, and risk of embolic events 1 2 3 . Herein, we report a case of successful complete obliteration of a large ascending colon varix and its contributors through a single-puncture endoscopic cyanoacrylate glue injection.

A cirrhotic patient with hematemesis and hepatic encephalopathy underwent endoscopic esophageal variceal ligation. Upon octreotide withdrawal, hematochezia occurred. Esophagogastroduodenoscopy was negative, while colonoscopy revealed a large lesion in the ascending colon bearing a rupture and clot ( Fig. 1 ). Hemostatic clips were placed as a temporary measure, octreotide was reintroduced, and computed tomography was performed, thus revealing a complex ascending colon varices network. A modified endoscopic cyanoacrylate glue injection, allowing safe single injection of larger volumes, was implemented ( Video 1 ). A 23G injection needle (Steris, Dublin, Ireland) was primed with 1 ml lipiodol (Guerbet, Chicago, Illinois, USA), the ascending colon varix was punctured near its base (targeting contributing vessels), and a 1-ml lipiodol plus 1-ml n-butyl-2-cyanoacrylate (Histoacryl; Β Braun, Melsungen, Germany) mixture was slowly (to avoid migration) injected while gradually directing the injection with upward scope flexion towards the varix apex (for complete occlusion). Saline (2 ml) was used to push the glue outside the needle, increase volume within the large varix, and accelerate polymerization. The needle was removed from the ascending colon varix and retracted while flushing. The protruding catheter (to avoid endoscope-related damage from the glue) was used to palpate the varix and confirm its stiffness. Subsequent X-ray confirmed complete occlusion of the ascending colon varices network ( Fig. 2 ).

**Fig. 1:**
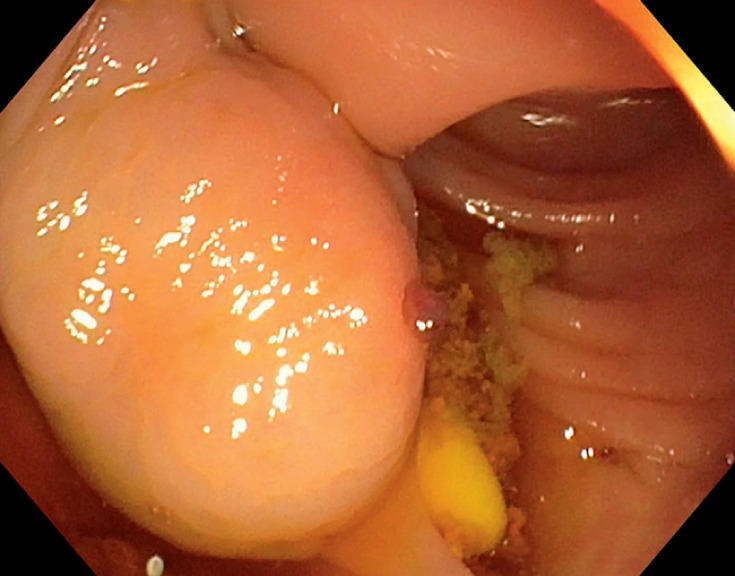
A large colonic varix bearing a small rupture and a clot was identified in the ascending colon.

Modified single-puncture endoscopic cyanoacrylate glue injection for efficient, safe, and complete obliteration of a bleeding ascending colon varix.Video 1

**Fig. 2:**
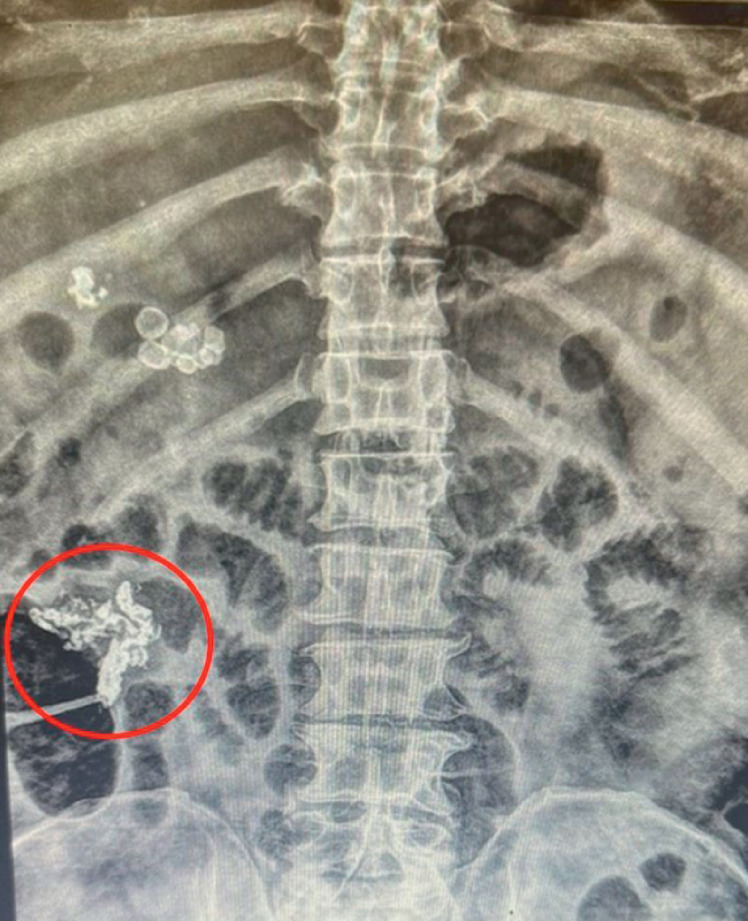
X-ray post-endoscopy confirms successful complete occlusion of the complex ascending colon varix and its contributors (red circle).

Bleeding from ascending colon varices can be managed with modified endoscopic cyanoacrylate glue injection, ensuring successful hemostasis and safe, complete obliteration of the varix through a single puncture.

Endoscopy_UCTN_Code_TTT_1AQ_2AZ

## Notes

Endoscopy E-Videos https://eref.thieme.de/e-videos
*E-Videos* is an open access online section of the journal *Endoscopy* , reporting on interesting cases and new techniques in gastroenterological endoscopy. All papers include a high-quality video and are published with a Creative Commons CC-BY license. Endoscopy E-Videos qualify for HINARI discounts and waivers and eligibility is automatically checked during the submission process. We grant 100% waivers to articles whose corresponding authors are based in Group A countries and 50% waivers to those who are based in Group B countries as classified by Research4Life (see: https://www.research4life.org/access/eligibility/ ). This section has its own submission website at https://mc.manuscriptcentral.com/e-videos .

## References

[1] Gulamhusein AF Kamath PS The epidemiology and pathogenesis of gastrointestinal varices Tech Gastrointest Endosc 2017 19 62 68

[2] Sunkara T Caughey ME Culliford A Idiopathic isolated colonic varices: an extremely rare condition J Clin Med Res 2018 10 63 65 10.14740/jocmr3230w 29238436 PMC5722047

[3] Karstensen JG Ebigbo A Bhat P Endoscopic treatment of variceal upper gastrointestinal bleeding: European Society of Gastrointestinal Endoscopy (ESGE) Cascade Guideline Endosc Int Open 2020 8 E990 E997 10.1055/a-1187-1154 32626821 PMC7329372

